# Receptors for Hyaluronic Acid and Poliovirus: A Combinatorial Role in Glioma Invasion?

**DOI:** 10.1371/journal.pone.0030691

**Published:** 2012-02-17

**Authors:** Zaynah Maherally, James R. Smith, Qian An, Geoffrey J. Pilkington

**Affiliations:** Cellular and Molecular Neuro-oncology Research Group, Institute of Biomedical and Biomolecular Sciences, School of Pharmacy and Biomedical Sciences, University of Portsmouth, Portsmouth, United Kingdom; City of Hope National Medical Center and Beckman Research Institute, United States of America

## Abstract

**Background:**

CD44 has long been associated with glioma invasion while, more recently, CD155 has been implicated in playing a similar role. Notably, these two receptors have been shown closely positioned on monocytes.

**Methods and Findings:**

In this study, an up-regulation of CD44 and CD155 was demonstrated in established and early-passage cultures of glioblastoma. Total internal reflected fluorescence (TIRF) microscopy revealed close proximity of CD44 and CD155. CD44 antibody blocking and gene silencing (*via* siRNA) resulted in greater inhibition of invasion than that for CD155. Combined interference resulted in 86% inhibition of invasion, although in these investigations no obvious evidence of synergy between CD44 and CD155 in curbing invasion was shown. Both siRNA-CD44 and siRNA-CD155 treated cells lacked processes and were rounder, while live cell imaging showed reduced motility rate compared to wild type cells. Adhesion assay demonstrated that wild type cells adhered most efficiently to laminin, whereas siRNA-treated cells (*p*<0.0001 for both CD44 and CD155 expression) showed decreased adhesion on several ECMs investigated. BrdU assay showed a higher proliferation of siRNA-CD44 and siRNA-CD155 cells, inversely correlated with reduced invasion. Confocal microscopy revealed overlapping of CD155 and integrins (β_1_, α_v_β_1_ and α_v_β_3_) on glioblastoma cell processes whereas siRNA-transfected cells showed consequent reduction in integrin expression with no specific staining patterns. Reduced expression of Rho GTPases, Cdc42, Rac1/2/3, RhoA and RhoB, was seen in siRNA-CD44 and siRNA-CD155 cells. In contrast to CD44-knockdown and ‘double’-knockdown cells, no obvious decrease in RhoC expression was observed in CD155-knockdown cells.

**Conclusions:**

This investigation has enhanced our understanding of cell invasion and confirmed CD44 to play a more significant role in this biological process than CD155. Joint CD44/CD155 approaches may, however, merit further study in therapeutic targeting of infiltrating glioma cells.

## Introduction

Local invasion of the brain by neoplastic glial cells is arguably the most significant biological feature of primary brain tumours which hinders successful therapy [1]. Glioma cell invasion of surrounding brain tissue frequently precludes complete surgical resection. Moreover, during this invasive procedure, cells transiently arrest from the cell cycle [2], therefore leaving them refractory to radiotherapy and the response to chemotherapy is accordingly poor [3]. The complex machinery of invasion involves key players including cell adhesion molecules, the extracellular matrix (ECM), gangliosides, growth factors and cytokines as well as the cytoskeletal elements [2].

ECM is central to the invasive behaviour of glioma cells, mediated through its associations with integrins [4]. In primary brain tumour, ECM is aberrantly overexpressed, providing a permissive substrate for invasion [3], [5]. Cell adhesion molecules (CAMs) such as integrins, cadherins and immunoglobulin proteins are involved in cell motility in response to soluble ECM proteins. Amongst them, integrins, consisting of α and β subunits [6], are key molecules on the cell surface which facilitate ECM/CAMs interaction and binding. Integrins play their mediating role in adhesive events during malignant transformation, tumour growth and progression, invasion and metastasis by providing a physical transmembrane link between the ECM and underlying cytoskeletal elements, which results in transduction of bidirectional signals across the cell membrane [4]. CAMs sit at the top of many signalling cascades that regulate actin and microtubule dynamics through Rho GTPases [7].

CD44, the CAM originally described as the lymphocyte homing receptor, is a polymorphic family of membrane glycoproteins [8]. CD44 is involved in a diverse range of physiological and pathological processes including lymphocyte homing and activation, cell-ECM interactions, migration, tumour growth and metastasis [9]. It has two isoforms with molecular weights of 80–90 kDa and 150 kDa. The former isoform aids the attachment to hyaluronic acid (HA), a component of ECM with relatively high concentration in the brain [10], [11] and also known to play a pivotal role in glioma invasion [12]. The role of HA in invasion has been described previously whereby cancer cells stimulate host cells to produce and secrete HA *via* a paracrine growth factor mechanism, known as HA stimulating activity [13]. In ECM, the secreted HA undergoes hydration and swells to extend the extracellular space, providing a pathway for neoplastic cell invasion. Invading cells can then adhere to HA by way of cell surface receptors including CD44 [14]. Having invaded into the new host tissue, invading tumour cells secrete hyaluronidase to breakdown HA molecules, giving rise to a pro-angiogenic product that facilitates continued survival of the neoplasm [5]. Previous data have shown that CD44 is overexpressed in neoplastic cells and aids glioma cell adhesion and invasion, through its interaction with HA and other ECM components [15]–[17].

CD155, a transmembrane glycoprotein originally discovered as the receptor for Poliovirus (PV), binds to vitronectin via its extracellular domain [18]. CD155, although widely distributed throughout human tissues, has a low level of expression that is tightly controlled. Increased expression of CD155 has been shown in tumours and recently Goetz *et al*. further reported an upregulation of CD155 on tumour cells in glioblastoma multiforme (GBM) patients. This study also underlines the possible association of CD155 with cancer stem cells which they have exploited in an attempt to use non-pathogenic poliovirus recombinant in preliminary therapeutic protocols [19]. Additionally, CD155 is thought to play a key role in tumour cell migration, enhancing cell adhesion signals and regulating focal adhesions [20]. It has been reported that CD155 is recruited to the leading edges of migrating cells where it co-localises with other migration mediators, namely actin and integrins [20]. Inhibition of CD155 by siRNA-knockdown (siRNA-KD) in cultured glioma cells alters cell morphology to a larger, more uniform phenotype with resultant significant reduction in cell migration/invasion [20], [21]. It was further stated by Enloe and Jay that inhibition of CD155 by RNAi caused a decrease in tumour invasion in part through reduced activity of matrix metalloproteinase-2 (MMP-2), a known factor in GBM invasion [22].

Interestingly, a physical association has been revealed between CD44 and CD155 on the cell membrane of monocytes where anti-CD44 monoclonal antibody (mAb) inhibits the binding of anti-CD155 mAb [23]. Similarly, anti-CD44 mAb hinders the binding of poliovirus to CD155 due to its steric position [24]. These studies reflect a close physical apposition between CD44 and CD155. It has been suggested that CD44 may act as a co-receptor for cellular uptake of poliovirus, however the localisation of CD44 in human tissues does not correlate with poliovirus susceptibility [24]. Such interaction between CD44 and CD155 and its implications have yet to be researched in brain tumours. In particular, the role of CD155 in brain tumours is still unclear and its potential as a therapeutic target for invasive brain tumours remains to be explored. In the present study, the juxtaposition of CD44 and CD155 was observed on glioma cells and their interactive role in cell migration/invasion was investigated in conjunction with integrins at the cell surface.

## Results

### Expression and close proximity of CD44 and CD155 on glioma cells

**Figure 1:**
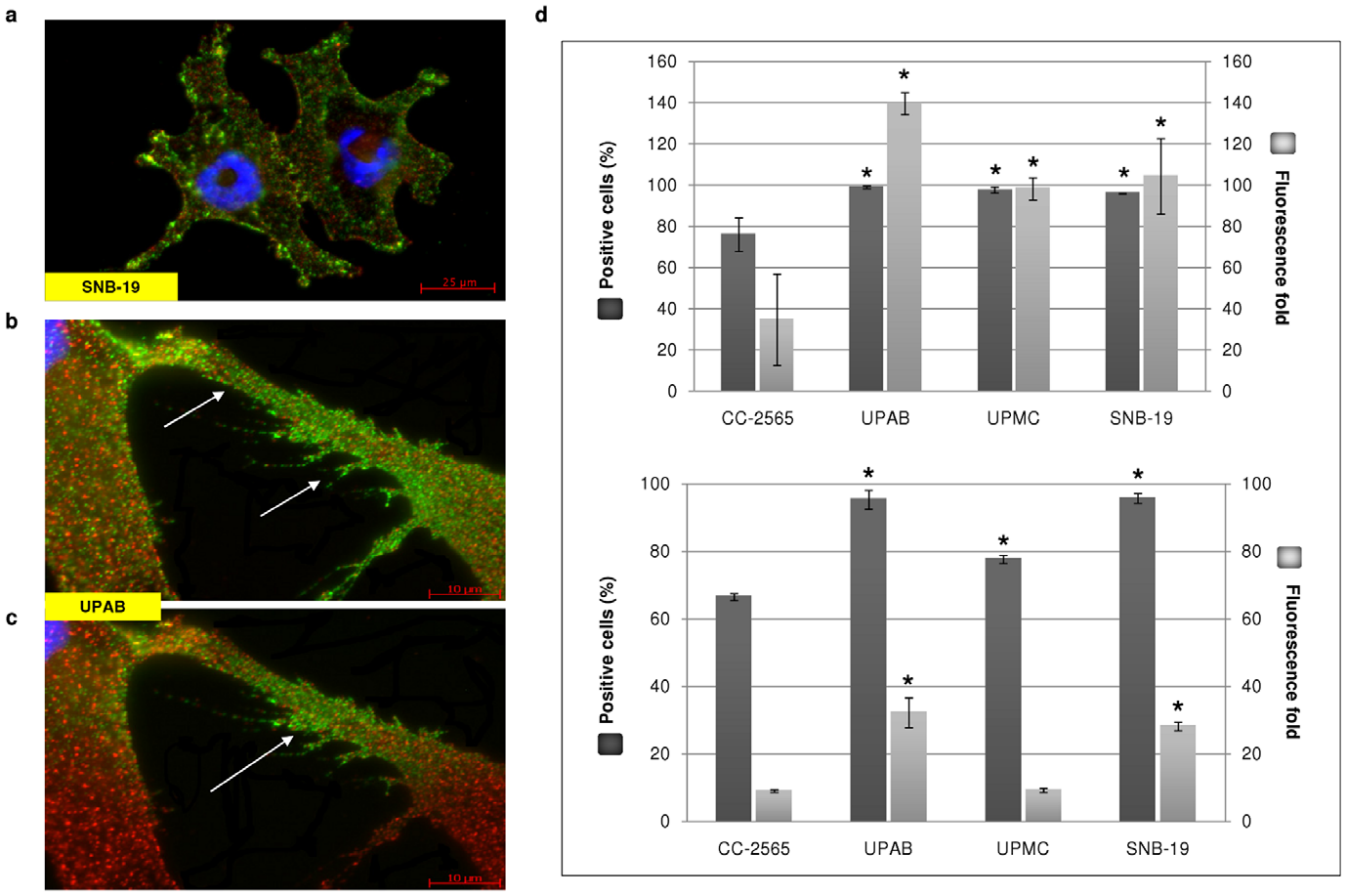
Expression and close proximity of CD44 and CD155 on GBM cells. **a–c**, Co-staining of CD44 (red) and CD155 (green) on SNB-19 and UPAB cells; scale bars: 25 µm (**a**) and 10 µm (**b**–**c**). TIRF microscopy (**c**) clearly demonstrated the juxtaposition of CD44 and CD155. CD155 is mainly expressed on the processes and invadapodia (arrows). **d**, Significantly increased expression of CD44 (**top panel**) and CD155 (**bottom panel**) in GBM cells confirmed by flow cytometry, as assessed by the percentage of antigen-expressing cells and increase in fluorescence fold. * indicates statistical significance compared to CC-2565, a normal human astrocyte cell line.

Expression of CD44 and CD155 on the surface of normal astrocytes and GBM cells were analysed by ICC and flow cytometry. CD44 was widely distributed over the cells but more condensed on the leading membrane and ruffles (Fig. 1a–c). CD155 was well distributed throughout the cells with more condensed expression at cell-cell junctions, established points of adhesion and the prominent filopodia (Fig. 1a–c). Total internal reflected fluorescence (TIRF) microscopy was used to clarify the exact location of CD44 and CD155, showing that CD44 and CD155 are closely physically apposed on the cell membrane (Fig. 1c). Compared with normal astrocytes (CC-2565), significantly higher expression of CD44 and CD155 in GBM cell lines was demonstrated by flow cytometry (*p*<0.0001 for both CD44 and CD155 expression in terms of positive cell population and fluorescence fold with the exception of UPMC which showed a similar fluorescence fold to CC-2565 (*p*>0.05; Fig. 1d).

### Evaluation of siRNA-KD of CD44 and CD155

**Figure 2:**
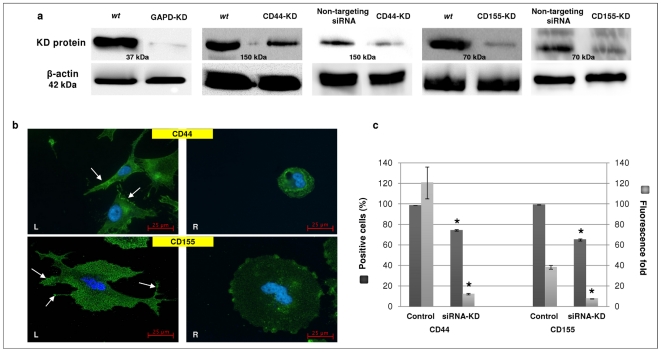
Reduced expression of CD44 and CD155 following siRNA-KD in SNB-19 cells. **a**, siRNA-KD of CD44 and CD155 was confirmed by Western blotting using both wild type (*wt*) and non-targeting siRNA treated cells as controls. GAPD-siRNA was used as a positive control to validate the knockdown effect. **b**, Expression of CD44 (**top panels**) and CD155 (**bottom panels**) in non-targeting siRNA treated cells (**control/left panels/L**) and relevant siRNA-KD (**right panels/R**) cells; scale bars: 25 µm. In control cells (**L**), CD44 is uniformly distributed with intense staining at the edges of the cells (arrows) whereas CD155 is well distributed with dense staining zones at the leading edges of the cells (arrows). CD44/CD155 staining was reduced in the siRNA-KD cells with clearly altered morphology (**R**). **c**, CD44/CD155-KD was confirmed by flow cytometry. Expression levels of CD44 and CD155 were significantly reduced in siRNA-KD SNB-19 cells as indicated by percentage of positive cells and fluorescence fold. * indicates statistical significance compared to non-targeting siRNA treated cells (control).

The effect of siRNA-KD was assessed by Western blotting, ICC and flow cytometry. SNB-19 was employed in the knockdown experiments. An Accell™ non-targeting pool siRNA was used as a negative control to demonstrate a non-specific baseline cellular response that could be compared to the levels in SNB-19 cells treated with target-specific siRNA (i.e., CD44 and CD155). Glyceraldehyde-3-phosphate dehydrogenase (GAPD)-siRNA was used as a positive control for gene silencing and validation of experimental design. Western blotting (WB) showed markedly decreased band intensity for GAPD and CD155, whereas CD44 band suggested residual protein present in treated cells, using wild type (*wt*) as well as non-targeting siRNA treated cells as controls (Fig. 2a). WB data for non-targeting siRNA treatment confirmed that sequence-specific knockdown of CD44 and CD155 was achieved. ICC staining revealed the decreased expression of CD44/CD155 and altered morphology of siRNA-KD cells (Fig. 2b). Non-targeting siRNA treated cells (Fig. 2b, left panel) showed well-defined, long processes extending at both ends, whereas the siRNA-KD cells assumed a round shape and had no processes (Fig. 2b, right panel). The degree of siRNA-KD was further quantified by flow cytometry. Significant decline of positive cell population and fluorescence fold was observed in siRNA-KD SNB-19 cells compared with control, e.g., non-targeting siRNA treated cells (*p*<0.0001 for both CD44 and CD155; Fig. 2c).

### Decrease in invasion and increase in proliferation of SNB-19 cells following CD44/CD155 monoclonal antibody (mAb) blocking and siRNA-KD

**Figure 3:**
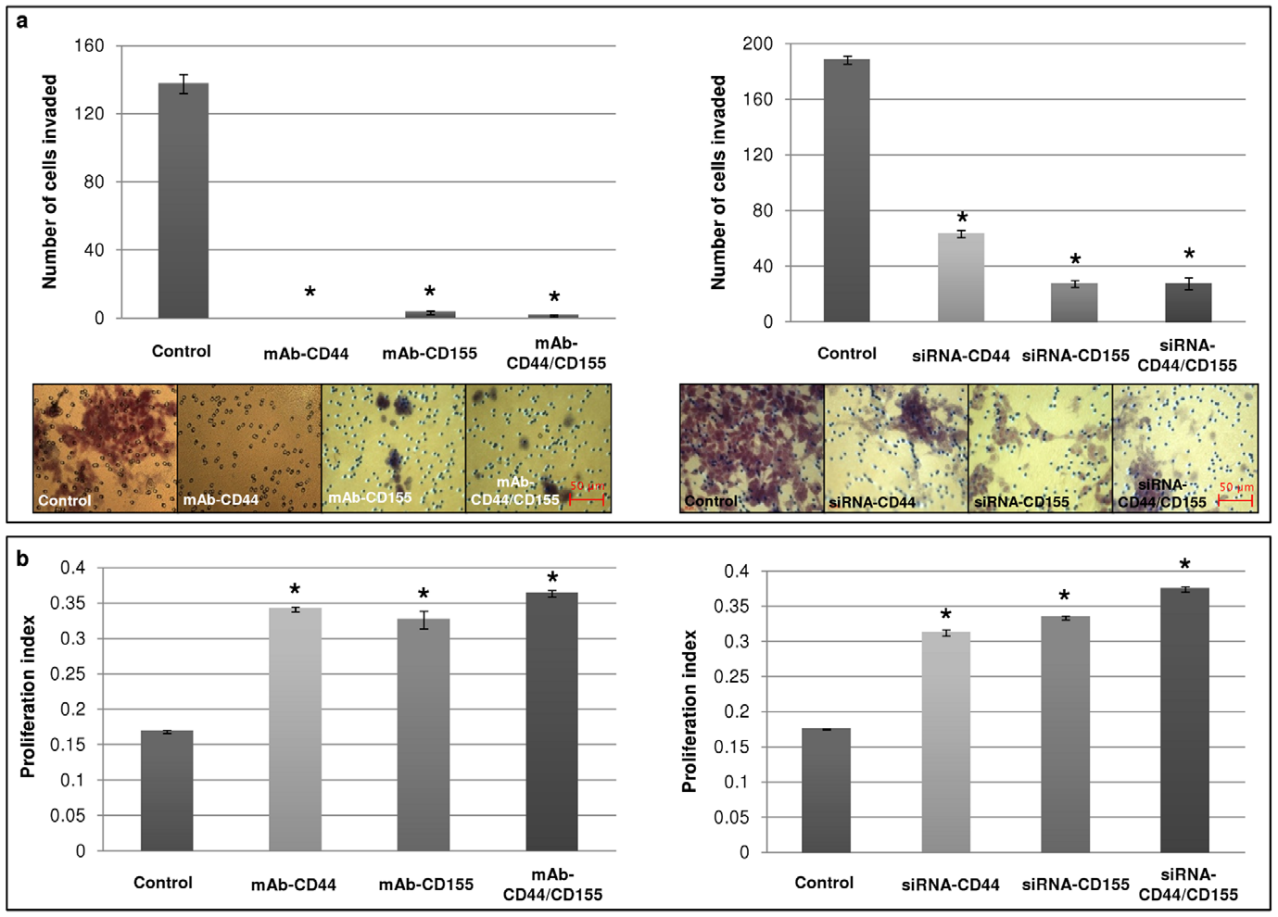
CD44/CD155-depletion inhibited invasion but enhanced proliferation of SNB-19 cells. Wild type (untreated) SNB-19 cells were used as a control in the mAb-blocking experiments; non-targeting siRNA treated SNB-19 cells were used as a control in the siRNA-KD experiments. **a**, Significantly reduced invasion following mAb-blocking (**left panel**) and siRNA-KD (**right panel**) was shown compared to controls. All images have scale bars of 50 µm. **b**, Proliferation was markedly increased in mAb-blocking (**left panel**) and siRNA-KD (**right panel**) cells compared to controls. * indicates statistical significance.

The role of CD44 and CD155 in glioma cell invasion was investigated by two approaches: mAb-blocking and siRNA-KD. Antibody-blocking of CD44 or/and CD155 significantly inhibited invasion of the GBM cells studied, compared with their wild type counterparts (control) (*p*<0.0001; represented by SNB-19 in Fig. 3a/left panel). In accordance with mAb-blocking, CD44/CD155-siRNA significantly reduced invasiveness of SNB-19 cells compared with non-targeting siRNA treated cells (control) (*p*<0.0001; Fig. 3a/right panel).

The BrdU cell proliferation assay was performed to determine the inverse relationship between invasion and proliferation as previously reported [25]. A significant difference was observed comparing manipulated SNB-19 cells with control cells. Wild type SNB-19 cells gave a proliferation index of 0.168, which increased to 0.341 and 0.326 in cells treated with mAb-CD44 and mAb-CD155, respectively. Further increase of proliferation rate was detected following simultaneous CD44/CD155-blocking, which gave a value of 0.363 (*p*<0.0001; Fig. 3b). Similarly, proliferation indices increased from 0.175 in non-targeting siRNA treated cells to 0.314, 0.333 and 0.375 in siRNA-CD44, siRNA-CD155 and ‘double’-KD cells, respectively (*p*<0.0001; Fig. 3b).

### Decrease in velocity of cell movement and total distance covered in CD44/CD155 siRNA-KD SNB-19 cells

**Figure 4:**
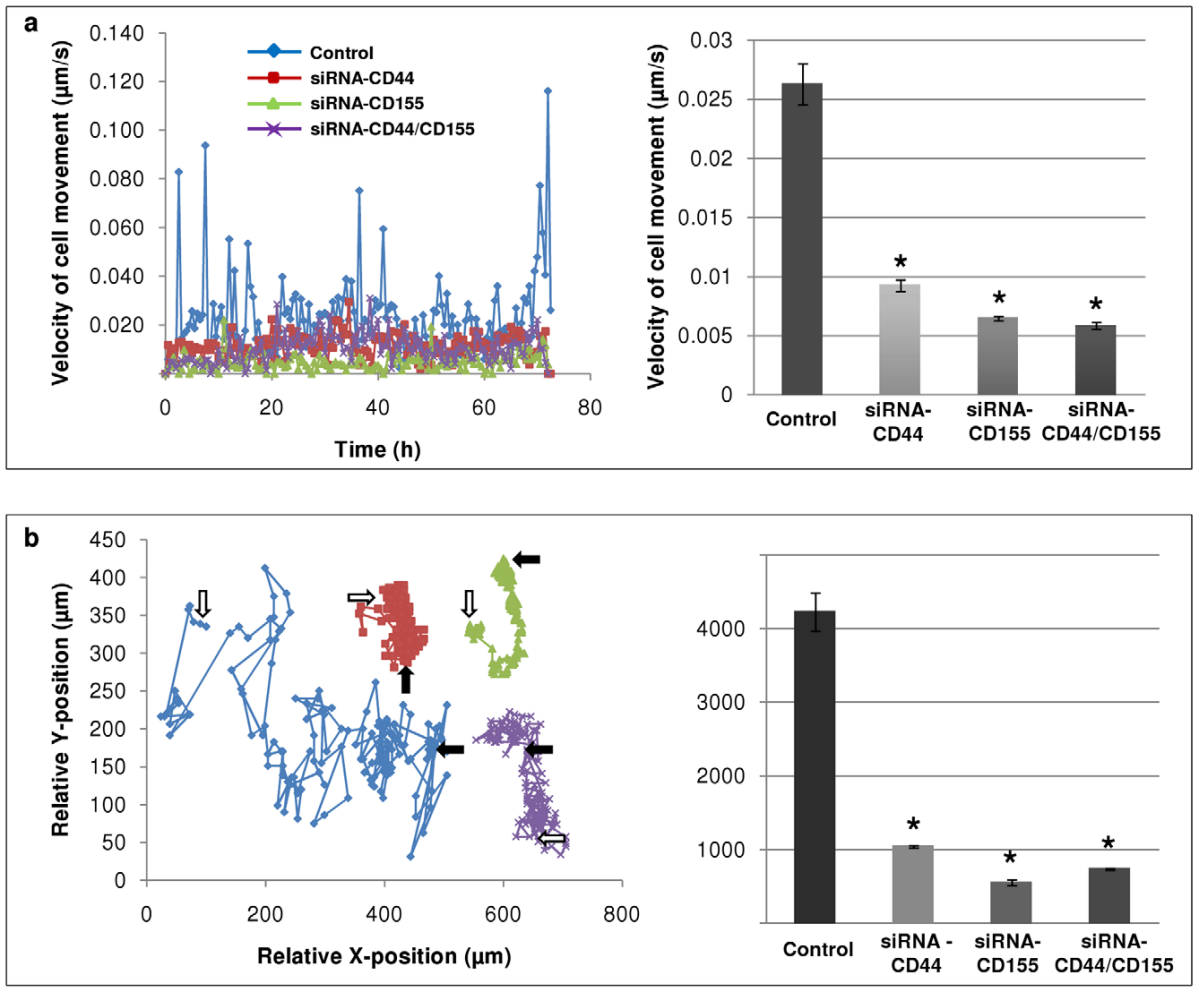
Reduced motility rate in CD44/CD155-KD SNB-19 cells. Non-targeting siRNA treated SNB-19 cells were used as a control. **a**, Velocity of cell movement (**right panel**) with cell tracking (**left panel**) analysed over a period of 72 h. The speed by which CD44/CD155-KD cells moved was significantly reduced. **b**, Total distance moved by cells (**right panel**) with cell tracking (**left panel**) over a period of 72 h. The distance travelled by CD44/CD155-KD cells was markedly decreased. Start position: white arrow; end position: black arrow. * indicates statistical significance compared to control.

CD44/CD155-KD SNB-19 cells were monitored by live cell imaging to evaluate motility over a period of 72 h. Control cells (non-targeting siRNA treated) showed the highest velocity of 0.026 µm/s. A marked decrease in velocity was noticed when cells were silenced for CD44 (0.0092 µm/s). siRNA-CD155 cells moved at a velocity of 0.0064 µm/s and the minimum velocity was achieved by ‘double’-knockdown cells (0.0058 µm/s). Our data suggest that CD44/CD155-KD significantly reduces glioma cell movement rate (*p*<0.0001) (Fig. 4a). The distance moved over 72 h was also significantly affected by CD44/CD155-KD (*p*<0.0001) (Fig. 4b). Control cells travelled the maximum distance of 4222 µm whereas siRNA-CD44 and ‘double’-KD cells moved only 1036 µm and 733 µm, respectively. A minimum distance of 549 µm was demonstrated by siRNA-CD155 cells.

### Decrease in adhesive potential of SNB-19 cells following mAb-blocking and CD44/CD155 siRNA-KD

**Figure 5:**
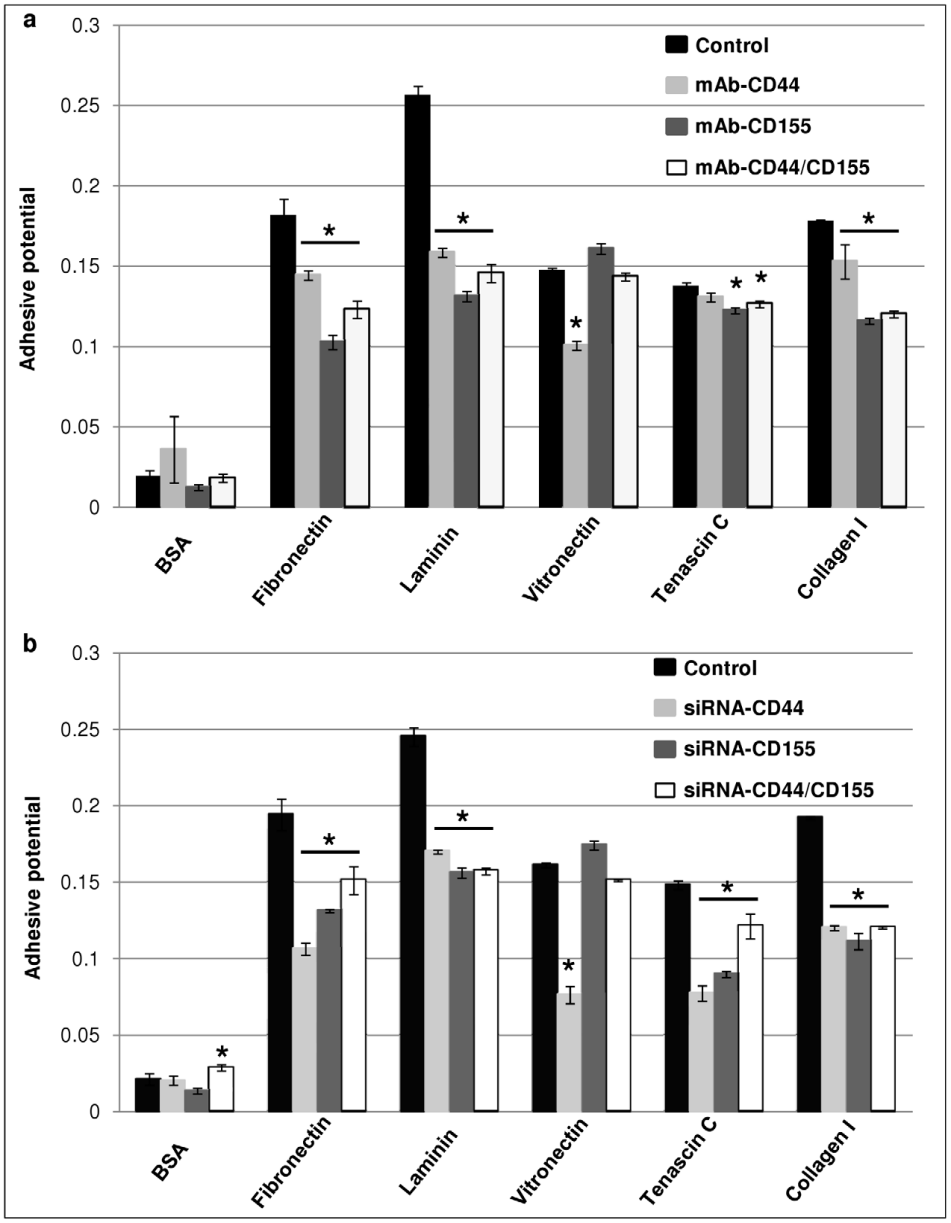
Decreased adhesive potential of SNB-19 cells following mAb-blocking (a) and siRNA-KD (b) of CD44/CD155. Five different human ECMs including collagen I, fibronectin, laminin, tenascin C and vitronectin were employed and BSA was used as the control substrate. For each ECM substrate, the adhesive potential of manipulated SNB-19 cells (*via* either mAb-blocking or siRNA-KD) was compared with the relevant control cells. Wild type SNB-19 cells served as a control in the mAb-blocking experiments whereas non-targeting siRNA treated SNB-19 cells were used as a control in the siRNA-KD experiments. * indicates statistical significance compared to control.

The interaction of cells with each of the adhesive substrates, *i.e.*, fibronectin, laminin, vitronectin, tenascin C and collagen I, was quantified for SNB-19 cells. Bovine serum albumin (BSA) was used as a control substrate. In the mAb-blocking experiments, control (wild type) and treated cells showed no significant difference for BSA (*p* = 0.442), while substantial decrease in adhesive potential was observed in the ‘single’- and ‘double’-blocking cells for fibronectin, laminin, tenascin C and collagen I compared with wild type cells (*p*<0.05; Fig. 5a), with the exception of mAb-CD44 in tenascin C treatment (*p*>0.05; Fig. 5a). Interestingly, unlike the trend shown by the above substrates, vitronectin demonstrated an increased interaction with mAb-CD155 treated cells (*p*>0.05; Fig. 5a), whereas the adhesive potential was reduced in mAb-CD44 and ‘double’-blocking cells compared with control cells (*p*<0.001 and *p*>0.05, respectively; Fig. 5a).

Unlike mAb-blocking, CD44/CD155 ‘double’-KD resulted in significantly enhanced adhesive potential compared with control SNB-19 cells (non-targeting siRNA treated) for BSA (*p*<0.05; Fig. 5b). Generally, in accordance with the results from the mAb-blocking experiments, ‘single’- and ‘double’-KD cells demonstrated significantly reduced interaction with fibronectin, laminin, tenascin C and collagen I compared with control cells (*p*<0.01; Fig. 5b). In the case of vitronectin, siRNA-CD44 cells showed the lowest adhesion rate (*p*<0.001; Fig. 5b), whereas siRNA-CD155 and ‘double’-KD cells showed insignificant change in adhesive potential compared with control (*p*>0.05; Fig. 5b).

### CD44 and CD155 knockdown is accompanied by changes in integrin expression

**Figure 6:**
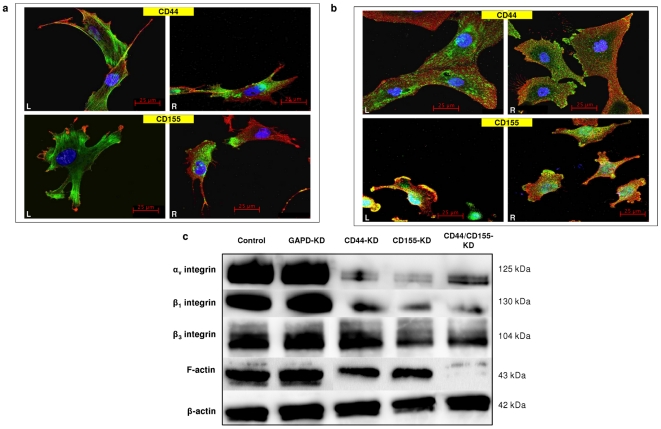
Expression of F-actin and integrins and their co-localisation with CD44/CD155 on GBM cells. **a**, Co-staining of F-actin (green/**left panels/L**) or β_1_-integrin (green/**right panels/R**) with CD44 (red/**top panels**) and CD155 (red/**bottom panels**) on wild type UPAB cells. **b**, Co-staining of α_v_β_1_-integrin (green/**L**) or α_v_β_3_-integrin (green/**R**) with CD44 (red/**top panels**) and CD155 (red/**bottom panels**) on wild type UPMC (CD44 staining) and non-targeting siRNA treated SNB-19 cells (CD155 staining). All images in **a** and **b** have scale bars of 25 µm. **c**, Western blotting showed reduced expression of F-actin and integrins (α_v_, β1 and β_3_) in CD44/CD155-KD SNB-19 cells when compared to non-targeting siRNA treated cells (control).

ICC double staining of CD44/CD155 and F-actin/integrins (β_1_, α_v_β_1_ and α_v_β_3_) was carried out with wild type UPAB, UPMC and non-targeting siRNA treated SNB-19 cells (representative confocal microscopy images shown in Figs. 6a & b). Intense filamentous, thread-like F-actin staining was noted within UPAB cells (Fig. 6a, left panels). Dense granular staining of β_1_-integrin was clearly seen at the perinuclear membranes and in the forming filopodia/invadopodia of the cell with some sparsely staining across the cytoplasm (Fig. 6a, right panels). Granular staining of α_v_β_1_-integrin was evenly distributed across UPMC and SNB-19 cells with more intense expression around the perinuclear region (Fig. 6b, left panels). α_v_β_3_-integrin was sprinkled all over the cell surface with aggregates at the end of the cell (Fig. 6b, right panels). CD44 was well distributed across the cells with prominent staining in the filopodia regions (Figs. 6a & b, top panels). CD155 staining was also evenly distributed with dense expression zones at the leading edges of the cells (Figs. 6a & b, bottom panels). A very low degree of CD155/β_1_-integrin co-localisation was noticed (Fig. 6a, right bottom panel) yet CD155/α_v_β_1_-integrin co-localisation was observed frequently around the peripheral edges of the cell (Fig. 6b, left bottom panel). A certain degree of random CD155/α_v_β_3_-integrin co-localisation was also seen on SNB-19 cell surfaces (Fig. 6b, right bottom panel). No obvious CD44/integrin co-localisation was observed by confocal microscopy.

A consequent decline in the expression level of integrins and F-actin was seen in CD44/CD155-KD SNB-19 cells (data not shown) and the consistent results were obtained through WB as demonstrated by the reduced intensity of bands compared with non-targeting siRNA treated cells (control) (Fig. 6c).

### CD44/CD15-KD is accompanied by changes in RHO GTPase signalling pathways

**Figure 7:**
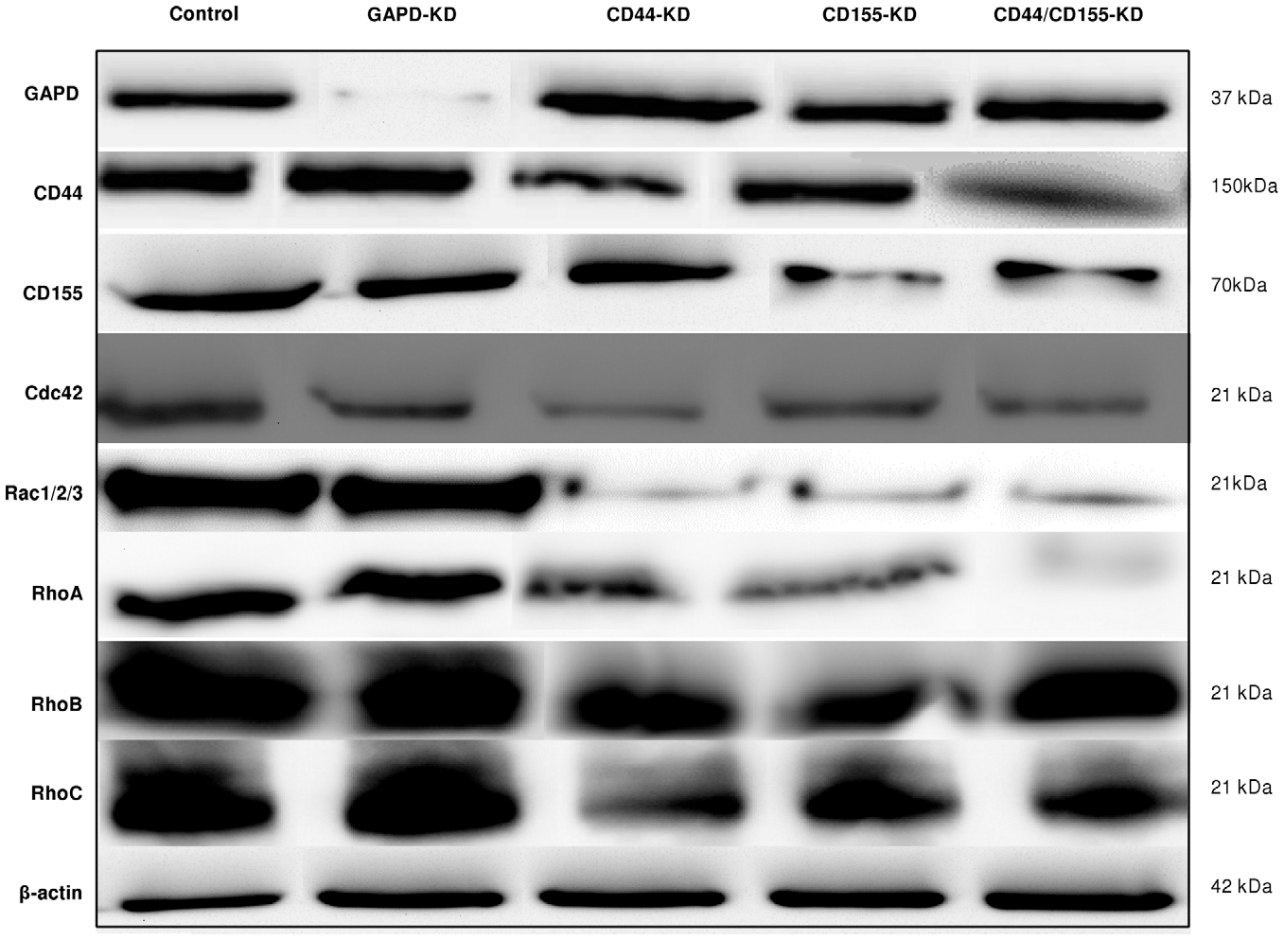
Western blotting of Rho GTPases in CD44/CD155-KD SNB-19 cells. High levels of Cdc42, Rac 1/2/3, RhoA, RhoB and RhoC were detected in non-targeting siRNA treated SNB-19 cells (control) whereas those proteins of interest were downregulated in CD44/CD155-KD cells.

To further investigate the role of CD44 and CD155 in cell invasion and the possible intracellular pathways they might regulate, certain key members of Rho GTPases (Cdc42, Rac 1/2/3, RhoA, RhoB and RhoC) were analysed in SNB-19 cells after CD44/CD155-KD. Western blotting detected high levels of the proteins of interest in control cells (non-targeting siRNA treated), whereas Rho GTPases were downregulated by CD44/CD155-KD (Fig. 7). Interestingly, CD44-KD had a greater impact on the expression of Cdc42 and RhoC compared with CD155-KD. Although Rac 1/2/3 and RhoA were downregulated similarly by siRNA-CD44 and siRNA-CD155, RhoA was nearly eliminated in ‘double’-KD cells, whereas Rac 1/2/3 level was similar to that in ‘single’-KD cells (Fig. 7). These data clearly indicate that CD44 and CD155 are involved in the intracellular signalling pathways. The level of CD44 and CD155 was also analysed by Western blotting to show whether silencing of one gene would alter the expression of the other gene. Our data suggest that CD44 and CD155 are not likely to regulate each other's expression (Fig. 7).

## Discussion

This study has revealed CD44 to exert a greater influence on glioma invasion than CD155. A possible synergistic role between the two receptors in the biological process, as suggested by their physical proximity [23] and previous studies demonstrating that blocking of CD44 impairs the receptor function of CD155 [24], was not confirmed.

In accordance with previous studies [15]–[17], [20]–[23], [26], our data show that CD44 and CD155 are highly expressed in glioma cells and are closely juxtaposed on the cell membrane. In the present study, CD44 was seen to be widely distributed over GBM cells but condensed on the leading edge of cells and processes, relating to its function as a CAM and attachment to HA. CD155 was also distributed throughout the cells with condensed expression at cell-cell junctions, adhesion points and on filopodia. These results are consistent with findings that CD155 is recruited to the leading edges of migrating cells and small areas of focal adhesion which are potentially involved in polarisation and directional motility [21], [27], [28].

Our previous studies have linked local invasion of glioma cells to elevated level of CD44 [29]–[31]. Similarly, CD155 expression and its interaction with ECM have been reported to play a key role in glioma cell adhesion, migration and invasion [20], [21], [32]. In the present study, upregulation of CD44 and CD155 was detected in three GBM cell lines (UPAB, UPMC and SNB-19) and a correlation between CD44/CD155 expression and cell invasiveness was observed, i.e., UPMC with the lowest CD44/CD155 level was less invasive compared with UPAB and SNB-19 (data not shown). However, the underlying mechanisms of those phenomena and the possible relationship between CD44 and CD155 in glioma cell invasion have remained unclear. In the present study, antibody-mediated blocking and siRNA-KD of CD44/CD155 in GMB cells were accompanied by decreased invasiveness, confirming the role of CD44/CD155 in cell invasion. CD44 showed a greater impact on cell invasion than CD155 as judged by the mAb-blocking and siRNA-KD studies. However, no additive or synergistic role of CD44/CD155 in curbing invasion was demonstrated. In accordance with previous reports, an inverse relationship between invasion and proliferation was also revealed in this study [26], [33]. Our data clearly demonstrate that CD44 and CD155 play important roles in glioma cell invasion.

To better understand the role of CD44/CD155 in glioma cell invasion, live cell imaging microscopy was applied to assess cell movement velocity and total distance covered by SNB-19 cells. The migration rate of SNB-19 cells was substantially reduced after CD44/CD155-KD with the lowest velocity observed in the ‘double’-KD cells. Interestingly, although total distance SNB-19 cells travelled in the period of 72 h was consequently reduced in CD44- and CD155-KD cells, the least distance was achieved by CD155-KD cells instead of ‘double’-KD ones, suggesting possible contribution of hampered directionality in CD155-KD cells. To our knowledge, this is the first time that CD44 and CD155 are shown to be able to promote an invasion-associated motility rate.

Additionally, morphological changes were observed in siRNA-CD44 cells, consistent with the published report that CD44-depleted cells are strikingly devoid of invadopodia [34]. This result therefore confirms that overexpression of CD44 correlates with glioma invasion [29]. CD155-silenced cells showed the same trend to become round, devoid of any invadopodia as previously shownby Sloan *et al.*, whereby CD155 depletion led to cell rounding up and absence of ruffles at the leading edges [20], [21]. Morphological changes following CD44- and CD155-KD were further supported by Western blotting data indicating significantly decreasedexpression of F-actin, integrins α_v_, β_1_, β_3_ and Rho GTPases. Whilst Rho is involved in the bundling of actin filaments into stress fibres and formation of focal adhesion complexes, Rac plays a role in the polymerisation of actin to form lamellipodia and membrane ruffles and Cdc42 is involved in the polymerisation of actin to form filopodia [35]. Thus, reduction in their expression renders the disorganisation of actin cytoskeleton and the inability of actin to form protrusions involved in cell invasion, leading to a more uniform and rounder morphology of cells.

Furthermore, adhesive potential was analysed in SNB-19 cells following mAb-blocking or siRNA-transfection. Both treatments caused a significant decrease in adhesion to different ECMs except vitronectin. Wild type SNB-19 cells adhered most effectively to laminin, followed by fibronectin, collagen I, vitronectin and tenascin C. A similar pattern was seen in both mAb- and siRNA-treated cells. Laminin is arguably the most important of all ECM proteins in the context of brain tumour invasion, while tenascin C is expressed in high quantities in human gliomas [5], [36]. The tenascins are thought to facilitate glioma cell migration by counteracting the adhesion activity of fibronectin to which they bind [36], [37]. The major ligand for CD44 is, however, hyaluronan [38], therefore HA/hyaluronan would have been the best ECM to assess the cell-ECM interaction. Unfortunately, it was not commercially available and thus attempting to produce HA in-house would have been costly and labour intensive. CD44 does, however, interact with other ECM components including collagen, fibronectin, laminin and fibrinogen [39]. CD155 binds specifically to vitronectin and the CD155/vitronectin interaction can be distinguished by a rapid complex formation [18]. Vitronectin binds to several cell receptors particularly of the integrin type, therefore plays a role in promoting cell attachment and invasion [4], [40]. Our results, however, show that CD155-depletion results in an enhanced cell adhesion to vitronectin, conflicting with the previously published data [21]. This could be due to the compensatory activity of CD44 and other adhesion mediators in the absence of CD155, as suggested by our data that CD44 is a much stronger receptor for vitronectin than CD155. These results can be further related to the marked decrease in cell velocity and distance travelled by SNB-19 cells under similar treatment regimes. This can be explained as if cells cannot adhere, they cannot move. Furthermore, no ECM coating was used in the live cell imaging assay, suggesting that glioma cells may produce their own autocrine stimulated ECMs [5].

CD155 co-localises with actin ruffles at the leading edges of migrating cells, suggesting a potential association between CD155 and actin cytoskeleton [20]. Previous studies have also revealed the co-localisation of CD155 with integrin subunit especially α_v_ and β_3_, known to mediate focal adhesion by forming physical transmembrane link between ECM and the underlying cytoskeleton elements [4], [41]. Therefore, CD155 expressed at the cell periphery and filopodia of migrating cells may be associated with adhesion and directional motility [20]. Integrins are involved in glioblastoma progression, with α_v_ and β_1_ being central to glioma invasion [42], [43]. In addition, integrin heterodimers α_v_β_1_ and α_v_β_3_ are receptors for vitronectin and are upregulated on glioma cells [44]. Moreover, CD44 mediates cell movement arrest and adhesion by promoting integrin activation [45], [46]. To elucidate the mechanisms underlying CD44/CD155-promoted glioma invasion, co-localisation of integrins or F-actin with CD44/CD155 was investigated. We found strong evidence that CD155 co-localises with α_v_β_1_- and α_v_β_3_-integrin at the leading edges of glioma cells. A consequent decline in the expression level of integrins and F-actin was seen following CD44/CD155-KD, accompanied by reduced co-staining of CD155/integrins α_v_β_1_ and α_v_β_3_. Our data suggest that CD155 plays a key role in glioma cell invasion *via* its interaction with critical integrin subunits.

The driving force for cell movement is provided by dynamic reorganisation of actin cytoskeleton, directing protrusion at the front of the cell and retraction at the rear [47]. The key regulators of actin cytoskeleton and adhesive structures are the Rho family of small GTPases (Cdc42, Rac and Rho) which play specific roles in cell motility and invasive phenotypes [37], [48], [49]. Previous studies have shown that CD44 is redistributed to lamellipodia and subsequently cleaved and shed from the cell surface when RhoA is over-activated in human glioblastoma cells (U251MG) [50]. Additionally, CD44 engagement induces Rac1 activation, cytoskeleton rearrangement and CD44 cleavage at the newly generated membrane ruffling areas during U251MG cell migration [51]. This raises the possibility that Rac1 activation upon HA/CD44/Rac1 engagement may trigger intracellular signals that induce CD44 cleavage and enhance tumour cell migration/invasion. In the present study, a marked decrease was observed in the expression level of Rac1/2/3 and RhoA in CD44/CD155-KD SNB-19 cells accompanied by curbed invasiveness. CD44-KD also caused obvious reduction in Cdc42 and RhoC expression. Based on previous and present studies, it can be postulated that CD44 and CD155 may play important roles in cell migration/invasion by engaging with Rho GTPases.

This study demonstrates the close proximity but not co-localisation of CD44 and CD155 on the cell surface. For the first time, CD44 and CD155 have been studied side-by-side to understand their roles in cell migration/invasion and their interaction with integrins and Rho GTPases. CD44/CD155-silencing significantly inhibited the invasive phenotype of glioma cells associated with decreased expression of some of the key mediators of invasion, particularly F-actin, integrins, Rac 1/2/3, RhoA and Cdc42. Our findings indicate that CD44 and CD155 are key players in glioma progression; CD44 playing a more significant role in this context. In order to further investigate the possibility of synergistic or co-operative role between CD44 and CD155 in glioma invasion (not evidenced in this study) we plan to carry out additional, in-depth studies of signal transduction pathways. Indeed, an intimate knowledge of interactive processes underlying invasion, *e.g.*, those involving CD44 and/or CD155, may prove valuable in the development of new therapeutic strategies.

## Materials and Methods

### Ethics Statement

Biopsies from patients with glioblastoma were obtained under Ethics permissions LREC 00-173 or KCH 11-094 or 11/SC/0048 in accordance with the National Research Ethics Service (NRES). All patients consented to the use of biopsy material for research purposes. Consent forms were read to and duly signed by participating patients prior to biopsy.

### Cells

Cell lines were cultured in Dulbecco's Modified Eagle Medium (Gibco) with 10% foetal calf serum (Sigma). The established human glioblastoma cell line, SNB-19 (passage-44), was obtained from the DSMZ German Brain Tumour Bank. Low-passage human glioblastoma cell lines, UPAB (passage-8) and UPMC (passage-7), were established in-house from biopsy-derived cultures. Normal human astrocytes, CC-2565 (Lonza), were used as controls.

### Antibodies

#### Primary antibodies

Mouse monoclonal anti-CD44 (1∶500 in immunocytochemistry; 1∶25 in flow cytometry) and anti-CD155 (1∶200; 1∶20) were purchased from Chemicon and R&D systems respectively. Blocking antibodies for CD44 (1∶100) and CD155 (1∶100) were obtained from Cambridge Biosciences. Mouse monoclonal anti-F-actin (1∶200) from Invitrogen and rabbit polyclonal anti-integrin β_1_ (1∶100), α_v_β_1_ (1∶100) and α_v_β_3_ (1∶200) from Abcam were used for immunocytochemistry staining. Mouse monoclonal anti-β-actin (1∶500) (Sigma), anti-integrin α_v_ (1∶250), β_1_ (1∶500) and β_3_ (1∶2500) (BD Biosciences) and goat polyclonal anti-GAPD (1∶200) (Abcam) were used for Western blotting.

#### Secondary Antibodies

Horseradish peroxidase (HRP)-conjugated IgG (Invitrogen) was used for chemiluminescent detection in Western blotting (1∶1000). Fluorochrome-conjugated AlexaFluor-488 and -568 (Invitrogen) were used in flow cytometry (1∶500) and ICC (1∶500).

### siRNA-KD of CD44/CD155 in glioblastoma cells

SMARTpool® siRNA (Dharmacon) targeting CD44 and CD155 were transfected into SNB-19 cells following the manufacturer's instructions. Cells were incubated with siRNA (100 µM) for 96 h to achieve >80% knockdown of CD44/CD155 and for 120 h in morphology assays. An Accell™ siRNA control kit (Thermo Fisher Scientific) was used to test specificity and stability of knockdown and efficiency of siRNA uptake by the cells, including an Accell™ non-targeting pool siRNA as the negative control and GADP-siRNA as the positive control. The non-targeting pool siRNA consists of 4 siRNAs with at least 4 mismatches to any human gene, designed not to target any genes in the human genome. It also distinguishes sequence-specific silencing from non-specific side effects with minimal impact on cell viability and cell phenotype. The GADP-siRNA serves to optimise and monitor efficiency of siRNA delivery into SNB-19 cells. All the siRNA transfection experiments were carried out at matching concentrations and incubation period.

### Immunocytochemistry (ICC)

ICC was performed according to the established protocols in the laboratory [52]. Briefly, cells were seeded onto sterile coverslips within a 6-well plate at 1×10^5^/well density and allowed to reach 70–80% confluency before immunostaining. Cells were fixed with 4% paraformaldehyde (PFA) (Sigma-Aldrich) and permeabilised with 0.2% Triton X-100 (Sigma-Aldrich) for intracellular antigen detection. Cells were then blocked with 10% serum/PBS (Sigma-Aldrich) and incubated with the relevant primary antibody, followed by secondary antibody incubation. Washes were carried out in PBS (5 min×3) before and after each antibody incubation and where double immunolabelling was performed, antibodies (raised in different hosts) were cocktailed for both incubation steps. Nuclei were counterstained with Hoechst Blue (Sigma-Aldrich) then coverslips were mounted with Vectashield® (Vector Laboratories). The slides were viewed using a Zeiss Axio ImagerZ1 fluorescence microscope. Images were captured using Volocity Image Analysis Software (V5.2, Perkin Elmer).

### Flow cytometry

Flow cytometry was carried out based on the standard protocols [52]. For intracellular antigen staining, cells were permeabilised with cytofix/cytoperm solution (BD Biosciences) at the beginning of the procedure. Cells were blocked in 5% serum/PBS (Sigma-Aldrich) prior to primary antibody incubation then washed with 5% serum/PBS and incubated with secondary antibody. After the incubation, cells were washed again and resuspended in 1% serum/PBS then transferred to Fluorescence-activated cell sorting (FACS) tubes (BD Biosciences). Shortly before analysis propidium iodide (PI) (Sigma-Aldrich) was added to samples in order to enable cell viability correction, except for intracellular antigen detection. Analysis was performed on a four-colour-multi-parameter FACS Calibur (BD Biosciences) equipped with a 488 nm argon gas laser and a 635 nm red diode laser. Each sample was analysed in triplicate plus one negative control (i.e. with primary antibody omitted) and the experiment was repeated three times. The expression level was assessed by the percentage of positive cell population (positive cells (%)) and the mean amount of antigen expressed by positive cells (fluorescence fold normalised against negative control) as described previously [52].

### Total internal reflected fluorescence (TIRF) microscopy

ICC specimens on microscope slides with coverslips (facing downwards) were examined with a Zeiss Aviovert 200 M (inverted) microscope incorporating a TIRF microscope (λ = 488 nm) on coverslips. Fluorescence was detected using excitation wavelengths of 488 nm (green), 568 nm (Red) and 405 nm (blue) with an argon ion laser. A 100× Plan-Apochromat oil immersion objective (working distance = 0.10 mm) with a high numerical aperture objective (NA>1.46) was used to obtain TIRF images. After the internal reflectance angle was identified, images were acquired using AxioVision software (V4.7, Carl Zeiss).

### Confocal microscopy

ICC images were captured using the 40× oil immersion objective of a Zeiss LSM 510 Meta Axioskop2 confocal microscope. Fluorescence was detected using excitation wavelengths of 488 nm (green), 568 nm (red) and 405 nm (blue), with an argon, HeNe1 and diode laser, respectively. Images were taken using optimal settings for pinhole diameter, detector gain and offset acquisition to detect positive immunofluorescence labelling with minimal background. Multi-track image capture was used with two channels so that separate channels could image different colours to help prevent any overlap in excitation spectra. Identical settings were then used to image negative controls in which primary antibody was omitted.

### Live cell imaging microscopy

Cells of interest were plated at 30% confluence in a 24-well plate and left to adhere overnight prior the experiment. The Zeiss Axiovert 200 M (inverted) live cell (time-lapse) microscope was then set-up to image 1 point in each well, once every 30 min over 72 h to monitor cell movement (37°C, 5% CO_2_, humid atmosphere). The images were collated and movie sequences generated. Cell tracking for the velocity and distance moved was enabled using the Volocity software (V5.4, Perkin Elmer). Ten cells were tracked randomly for each well and each experiment was done in triplicate and repeated three times. Non-targeting siRNA treated SNB-19 cells were used as the control.

### Invasion assay

Cell invasion was assessed by the ‘Transwell’ modified Boyden chamber technique [52]. Briefly, invasion was allowed to occur for 6 h in the incubator. A time-point analysis (2 h, 4 h, 6 h, 8 h, 10 h, 12 h) was carried out before choosing ‘6 h’ as the optimal incubation time for the invasion assay which was further supported by our previous studies [26]. This provided enough time for SNB-19 cells to invade prior proliferation based on the population doubling time calculated for this cell line. Non-invading cells on the upper surface of the filter were removed and invaded cells, adherent on the lower filter surface, were characterised through alkaline phosphatase vector red (Vectorlabs) staining. Images were captured by a Zeiss Axiophot brightfield microscope using the AxioVision software (V4.4, Carl Zeiss) and cells were counted in 5 random fields. For the mAb-blocking treatment, wild type SNB-19 cells were used as control and cells were incubated with 100 mg/ml of the antibody during the invasion period. For the siRNA-KD assay, non-targeting siRNA treated SNB-19 cells were used as control. Each experiment was performed in triplicate. Invasion was expressed as mean ± SEM of the number of total cells counted per well.

### Proliferation assay

The Bromodeoxyuridine (BrdU) Cell Proliferation Assay Kit was used to assess the proliferation rate of the cells according to the manufacturer's protocol (Chemicon). The 96-well plate was read on a spectrophotometer microplate reader (POLARstar OPTIMA BMG Labtech). The proliferation index was reflected by the optical density (OD) reading. All tests were done in triplicate and repeated three times. Controls used in mAb-blocking and siRNA-KD assays were the same as those in the invasion assay (see above).

### Adhesion assay

The ECM Cell Adhesion Array Kit (Chemicon) was used for adhesion assay. The 96-well ECM Array plate consists of wells coated with five different human ECMs (collagen I, fibronectin, laminin, tenascin C, vitronectin) and wells coated with BSA which serves as the control substrate. Cells were incubated for 2 h then unbound cells were washed away. Adherent cells were lysed with NaOH (1 M) and detected by the CyQuant GR® dye. Relative cell attachment was determined using the same microplate reader as above. All tests were carried out in triplicate and repeated three times. Control cells used in mAb-blocking and siRNA-KD assays were the same as those in the invasion assay (see above).

### Western blotting (WB)

WB was performed according to a standard protocol. Briefly, cell lysates were separated in 10% acrylamide SDS-PAGE gel and proteins were detected using the primary antibodies and horseradish peroxidase-conjugated secondary antibodies. Immunocomplexes were revealed using an enhanced chemiluminescence reagent (Cheshire Sciences) and light sensitive films (Amersham). The blot was visualised and analysed with the GBOX Chemi XT16 system (Synoptics). Wild type and non-targeting siRNA treated cells were used as controls in various assays (refer to individual result and figure legend). For siRNA-KD experiments, all transfected cells were harvested after 96 h siRNA-incubation for lysate extraction.

### Rho GTPases signal transduction pathway

The Rho-GTPase Antibody Sampler Kit (Cell Signaling Technology) was used to investigate the effects of siRNA-CD44 and siRNA-CD155 on actin cytoskeleton reorganisation in SNB-19 cells. Western blotting was performed to detect the proteins of interest, as described above, using non-targeting siRNA treated SNB-19 cells as a control. The antibodies used are as follows: anti-Cdc42 (1∶250), Rac1/2/3 (1∶500), RhoA (1∶500), RhoB (1∶500) and RhoC (1∶500). All antibodies were rabbit polyclonal.

### Statistical analysis

Statistical analyses were performed using one-way ANOVA followed by Tukey's multiple comparison post test with *p*<0.05 being regarded as significant. The software package GraphPad Prism 3.02 was used and all data are presented as mean values.
